# Role of Serologic and Molecular Diagnostic Assays in Identification and Management of Hepatitis C Virus Infection

**DOI:** 10.1128/JCM.02407-15

**Published:** 2016-01-28

**Authors:** Gavin Cloherty, Andrew Talal, Kelly Coller, Corklin Steinhart, John Hackett, George Dawson, Juergen Rockstroh, Jordan Feld

**Affiliations:** a Abbott Diagnostics, Abbott Park, Illinois, USA; b University at Buffalo, Buffalo, New York, USA; c ViiV Healthcare, Research Triangle Park, North Carolina, USA; d University Bonn, Bonn, Germany; e University of Toronto, Toronto, Canada

## Abstract

The drugs available for the treatment of hepatitis C virus (HCV) have evolved to provide shorter treatment duration and higher rates of sustained virologic response (SVR), and the role of HCV infection diagnostic tests has had to evolve in order to meet changing clinical needs. This review gives an overview on the role of HCV infection diagnostic testing (molecular and serological tools) used in the diagnosis and management of HCV infection. All of this critical information guides physician decisions to optimize patient clinical outcomes. Also discussed is the future direction of diagnostic testing in the context of further advances in drug development.

## INTRODUCTION

It is estimated that 130 to 170 million people worldwide are chronically infected with the hepatitis C virus, with 3 to 4 million new infections per year and over 350,000 deaths due to hepatitis C virus (HCV)-related liver disease each year (1). The long-term impact of HCV infection is highly variable, ranging from minimal effects to chronic hepatitis, advanced fibrosis, cirrhosis, and hepatocellular carcinoma (HCC) (2). The development of detection assays for HCV has paralleled that of our understanding of the infection and the introduction of increasingly effective therapies with progressively decreasing adverse effects. Serologic and molecular assays for HCV have played major roles in the identification of those with the viral infection, in determining the severity of the disease, and in the response to therapeutic interventions. In this article, we focus on the changing role of these assays, particularly in light of recent advances in the mandate for HCV screening and the changing therapeutic landscape.

## ROLE OF TESTING IN HCV INFECTION DIAGNOSIS AND SCREENING THE BLOOD SUPPLY

Unsafe medical practices, including unregulated blood transfusions without effective screening, as well as illicit injection drug use have historically been major drivers of viral hepatitis infections. U.S. investigators who retrospectively studied blood donor and recipient repositories collected from the 1960s subsequently found that 25% of recipients had been infected with HCV (3). These findings prompted a pivotal switch to an all-volunteer blood donor system in 1970, which, coupled with the exclusion of hepatitis B surface antigen-positive blood, dramatically reduced transmission of HCV through transfusion to 7% (4, 5). Since the identification of the virus through molecular methods in 1989, our understanding of the biology of HCV has permitted the development of HCV-specific diagnostic assays; their application to diverse clinical samples has elucidated routes of viral transmission. Subsequently, public health efforts continued to focus on the reduction in the number of incident infections through the development and continual improvement of HCV-specific enzyme immunoassays (EIAs), which, combined with the recombinant immunoblot assay (RIBA) for confirmation, drove the incidence of transfusion-related HCV to less than 0.01% by the end of the 1990s (6).

One drawback of screening for antibodies to HCV is a potential delay in diagnosis of the infection, since HCV seropositivity may occur several weeks to months after virus exposure. This “preseroconversion window,” which is defined as the time interval when HCV RNA or core antigen (cAg) may be detected without detection of anti-HCV antibodies, resulted in blood bank adaptation of nucleic acid-based testing (NAT) in the late 1990s. This practice shortened the window period for an individual donor from up to 13 weeks with EIA-based testing to 3 days with NAT-based assessment. Consequently, the current risk of transfusion-related HCV infection is 1 per million units transfused (0.0001%) (7).

Similar decreases in injection-associated HCV transmission occurred with the introduction of harm reduction techniques principally meant to decrease HIV transmission (8). Initiated in most high-income countries around 1995, large-scale HIV prevention programs, including syringe exchange, pharmacy sales of injection equipment, and expanded methadone treatment, resulted in very large reductions in HIV incidence. The reduction in HCV transmission among persons who inject drugs (PWID), however, has been much less than that achieved with HIV since HCV is more readily transmissible (9). Sharing preparation equipment and a higher frequency of sharing are more likely to transmit HCV than HIV, resulting in a higher HCV prevalence among PWID. While the overall incidence of HCV infection in the United States has decreased as a result of improved blood donation and harm reduction practices, the HCV incidence has been increasing among certain segments of the population, namely, young, nonurban, largely white PWID (10). Many incident HCV infections have their origin among those who initially abused prescription opioids, with first use occurring, on average, 2 years prior to initiating heroin and sharing of injection needles. These findings suggest that expansion of treatment for substance use disorders, harm reduction techniques, and safe injection practices is required to terminate incident HCV infections.

Besides incident infections, an estimated 45% to 85% of subjects with prevalent HCV infections in the United States, whether transmission occurred through blood products, injection drug use, or other means, are unaware of their HCV infection status (11). In resource-limited settings, diagnosis rates are even lower. Since the highest HCV prevalence (76.5%) occurred among individuals born between 1945 and 1965 and since individuals in this birth cohort account for the majority of those who are unaware of their diagnosis, the Centers for Disease Control has recommended one-time serologic testing for all people born between 1945 and 1965 (12).

## ROLE OF TESTING IN DIAGNOSIS OF ACUTE HCV INFECTION

The diagnostic methods associated with treatment are different from those designed to screen and protect the blood supply. As treatments of HCV have improved dramatically in recent years, the diagnostic paradigm has also evolved to meet the changing clinical needs. Approximately 15% to 30% of patients spontaneously clear HCV within 6 to 12 months of initial exposure. Such individuals remain anti-HCV antibody positive but have no detectable viremia and are truly cured of the infection, without long-term sequelae. Prior to placing a patient on therapy, a physician must differentiate those people who have been exposed to HCV but who have, after acute and usually asymptomatic infection, resolved their infection without sequelae from those who have been exposed and have progressed to a chronic and potentially lifelong infection (13). Traditionally, the RIBA was used to confirm HCV infection by demonstrating reactivity of antibodies in the serum with specific HCV proteins, increasing the specificity of the initial antibody enzyme-linked immunosorbent assay (ELISA). However, this test has been discontinued and is consequently no longer available. The presence of HCV RNA in the peripheral blood is a reliable marker of HCV replication and is the main marker used today to confirm an active infection. Two technologies are routinely used for the qualitative HCV RNA test, PCR and transcription-mediated amplification (TMA).

**FIG 1:**
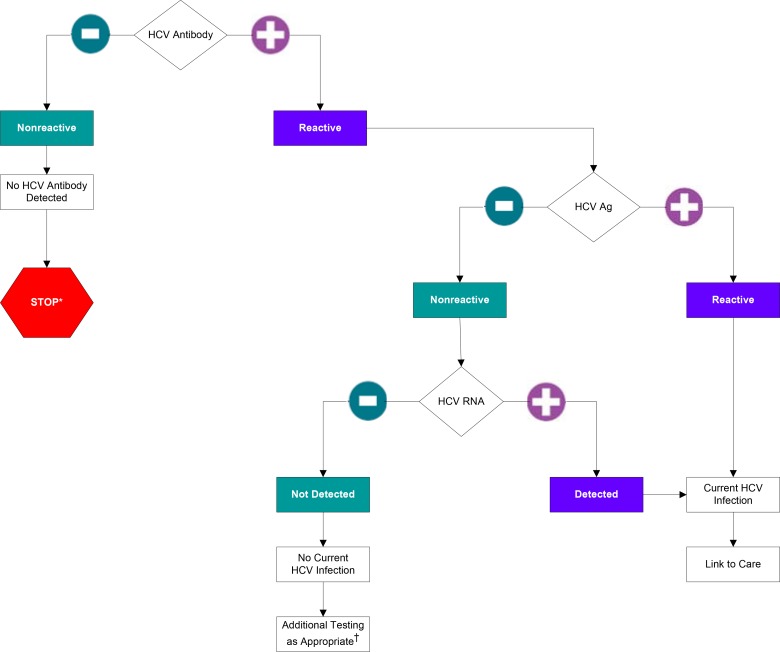
Algorithm for the use of HCV Antigen assay in conjunction with the HCV RNA test for the accurate identification of actively replicating HCV based on the findings of this review and previously published work. *, for persons who might have been exposed to HCV within the past 6 months, testing for HCV RNA for follow-up testing for HCV antibody is recommended. For persons who are immunocompromised, testing for HCV RNA can be considered. †, to differentiate past, resolved HCV infection from biologic false positivity for HCV antibody, testing with another HCV antibody assay can be considered. Repeat HCV RNA testing if the person tested is suspected to have had HCV exposure within the past 6 months or has clinical evidence of HCV disease or if there is concern regarding the handling or storage of the test specimen.

An alternative to directly measuring HCV RNA is quantification of the amount of HCV proteins in the blood. Production of HCV proteins requires viral replication and thus serves as a reliable marker of active infection. HCV core antigen testing has been the most extensively validated and the only commercially available approach for detecting HCV proteins in serum or plasma, although this test is available in the United States for research use only. Studies performed with different HCV core antigen tests have shown that, while assays using core antigen levels are not as sensitive or precise as measurement of HCV RNA levels, those levels are significantly related to the HCV RNA level (14). Because HCV core antigen analysis is faster and usually less expensive than NAT approaches, there is increasing interest in using this test as a reflex test for HCV-seropositive samples to identify individuals who are actively infected with HCV, particularly in resource-limited regions. Due to the lower sensitivity of HCV core antigen tests compared to HCV RNA tests, in order to achieve 100% detection of active viremia, confirmation of HCV RNA negativity should be considered in HCV-seropositive samples with negative HCV Ag results (Fig. 1). As the number of cAg false-negative results would be expected to be very low in the general population, the number of RNA tests required to confirm cAg negative results could be reduced while maintaining the 100% detection goal by pooling samples prior to HCV RNA testing. These numbers could be further reduced by triaging/stratifying samples for confirmation on the basis of those with an elevated aminotransferase level.

Generally, three separate populations are at risk for acute HCV infection: PWID (representing the major transmission risk in developed nations), patients at risk for nosocomial acquisition (usually in developing nations), and HIV-positive men who have sex with men (MSM) (15). With regard to MSM and, in particular, HIV-infected MSM, acute HCV infection is considered a sexually transmitted disease (16).

## ROLE OF DIAGNOSTIC TESTING IN TREATMENT OF CHRONIC HCV INFECTION

In contrast to many other viruses, HCV does not integrate into the host genome or persist in latent reservoirs in the body and as such is considered “curable,” in the context of antiviral treatment, termed a sustained virologic response (SVR). SVR is defined as undetectable HCV RNA following a defined period (usually 12 [SVR12] or 24 weeks) after therapy ends. The goal of therapy is to eradicate HCV infection in order to prevent complications associated with HCV-related liver disease (2).

Initially, therapeutic advances in the treatment of HCV infections were modest at best, with the use of interferon (IFN) being the cornerstone of HCV therapy; different IFN formulations have included interferon alfa and pegylated-interferon (Peg-IFN) alfa-2a or -2b alone or in combination with ribavirin (Peg-IFN/RBV). Although a cure of HCV infection has been achievable for many years, the reliance on interferon-based therapy, with its adverse side-effect profile and low cure rate, markedly limited treatment uptake. More recently, considerable investment in research and development has produced a large number of well-tolerated direct-acting antivirals (DAAs) and host-targeting agents (HTAs). In 2011, first-generation DAAs boceprevir and telaprevir, which are both HCV nonstructural 3/4A (NS3/4A) protease inhibitors, were approved for use in combination with Peg-IFN and RBV. More recently, combinations of DAAs have been developed with reported >90% cure rates in phase II and III trials, many of which were conducted with or without the use of IFN alfa and/or ribavirin.

In addition to confirming viremia, assays of the HCV RNA level and viral genotype and subtype are important tools to help physicians make treatment decisions. Prior treatment history, possibly including baseline resistance testing, is also helpful to guide therapy, as well as knowledge of the degree of liver injury and, possibly, of the interleukin-28B (IL-28B) genotype (at least in cases of IFN-containing HCV regimens), a marker of innate immune function. Treatment guidelines make recommendations as to how new therapies should be applied. The new HCV therapies have specific indications, including durations and drug combinations appropriate for use for treatment of a particular patient, which vary according to HCV genotype, a critical determinant of treatment response. The distribution of genotypes varies by geographic region and strongly influences the response to treatment. With interferon-based therapy, genotypes 1 and 4 proved less responsive than genotypes 2 and 3. However, with new interferon-free DAA regimens, genotype 3 is proving to be the most challenging to eradicate, although this may change with the approval of newer drugs. It is likely that genotyping will continue to be performed even as more pan-genotypic regimens are developed.

Subtyping of HCV genotypes has also proven important due to the lower barrier to resistance of genotype 1a isolates compared to genotype 1b for multiple classes of DAAs. This difference was initially seen with protease inhibitors but has also been shown to be relevant for nonnucleotide polymerase inhibitors and nonstructural 5A (NS5A) inhibitors. As an example, ribavirin is added to the treatment regimen for use in combination with AbbVie's recently approved paritaprevir-ombitasvir-dasabuvir in cases of HCV genotype 1a infection but not in cases of genotype 1b infection. The clinical significance of viral genetic diversity and subtype has been most extensively studied in pharmaceutical clinical trials focused on HCV genotype 1 infection. More studies on the impact of genetic diversity in other genotypes may reveal similar nuances which may further improve clinical outcomes. In wealthy countries, HCV genotype testing can now be performed in most clinical and hospital laboratories. A wide variety of genotyping methods are used, including PCR amplification followed by strip-based reverse hybridization, PCR followed by Sanger sequencing, and real-time PCR.

These various genotyping methods offer certain benefits, but they also have their limitations. To date, there have been no perfect genotyping tests. While sequencing can offer excellent resolution of HCV genotype, it can be time-consuming and labor-intensive, it requires skilled technologists and is costly, and results are not standardized. Real-time PCR methods offer workflow advantages, with a high degree of automation associated with reduced hands-on time, reduced technical expertise requirements, and reduced costs, and commercial methods which are approved for use in the United States (FDA) and Europe (CE) are now available. However, studies have shown that certain rare viral subtypes may not be resolved or accurately discriminated by this technology (17). Finally, strip-based reverse-hybridization methods are cheap but are manually performed and labor-intensive and result interpretation is subjective (18).

Interferon-based therapies have substantial side-effect profiles and only moderate efficacy (50% in HCV genotype 1 versus 75% in HCV genotypes 2 and 3) as measured by SVR 24 weeks post-treatment cessation. For these therapies, certain baseline predictors, such as a viral load of <800,000 IU/ml, infection with HCV genotype 2 or 3, and favorable patient genotype for certain genes associated with immune response such as the IL-28B gene, are considered valuable predictors of long-term treatment success. In areas of the world where interferon-based therapies continue to be prescribed, physicians need to weigh these predictors against patient clinical presentation in their decision of whether or not to initiate a course of interferon-based therapy. Once a patient has been started on an interferon-based regimen, monitoring of viral load and kinetics (for the approach known as response-guided therapy, or RGT) has proven to be very effective in guiding decisions regarding the duration of therapy to optimize rates of SVR (19).

The use of HCV kinetics to guide therapy has highlighted the need to standardize clinical thresholds and/or therapy guidelines to work across different assays for the measurement of viral load (20). In all the trials used to define the new RGT algorithm, a single test, the manual Roche High Pure system/Cobas TaqMan assay (HPS; Roche Molecular Systems Inc., Pleasanton, CA, USA) was used to measure HCV loads. Factors not taken into consideration during the development of the RGT algorithm were the results that might have been obtained with HCV RNA assays other than those used in the trials, such as Roche Cobas/Cobas TaqMan, Siemens kPCR, Abbott RealTi*m*e (ART), and the Qiagen Artus assay, which are all commercially available internationally and used in routine clinical practice. External studies were required to investigate how commutable these RGT rules were to other commercially available tests (20, 21).

In 2013, additional DAAs were approved for the treatment of HCV infections in Europe and the United States. The first was simeprevir, a second-generation protease inhibitor, followed shortly thereafter by sofosbuvir, the NS5B nucleotide polymerase inhibitor, representing a new class of drug. For both of these new drugs, prior knowledge of the viral genotype is still critical. In combination with Peg-IFN/RBV, simeprevir exhibited enhanced binding affinity and specificity for NS3/4A compared with the first-generation protease inhibitors indicated for the treatment of treatment-naive or -experienced patients infected with HCV genotype 1. In contrast, sofosbuvir was the first drug that allowed an interferon-free regimen with ribavirin for genotype 2 and 3 patients. After its approval, it became first-line therapy for genotype 1 and 4 patients when it was initially prescribed as a 12-week combination regimen with Peg-interferon and ribavirin. Patients ineligible for interferon could be offered a 24-week regimen of sofosbuvir and ribavirin.

In light of the stronger antiviral activity of DAAs compared with IFN-based therapy and the need to simplify treatment approaches, there has recently been a move away from Peg-IFN/RBV. Recent phase II and II IFN/RBV-free trials have eliminated the use of RGT: sofosbufir/daclatasvir administered for 12 or 24 weeks gave 100% SVR even without use of RBV. In the sofosbuvir/ledipasvir LONESTAR trial, 95% of patients achieved SVR whether treated for 8 or 12 weeks and, in the 12-week arm with RBV administration, the SVR rate was 100% (22). Additionally, the very high cost of DAAs and the desire to maximize adherence with as short a treatment duration as possible have raised the prospect of obtaining an SVR with as little as 6 or possibly even 4 weeks of antiviral therapy. Careful on-treatment kinetics may be useful in determining if these shorter duration therapies might be useful, although further studies are needed to evaluate this approach.

## ROLE OF VIRAL RESISTANCE TESTING IN HCV TREATMENT

The role of viral resistance testing in patients treated with DAAs remains largely unknown. In patients treated with simeprevir, in addition to viral genotyping, HCV genotype 1a-infected patients require antiviral drug resistance screening, as the presence of the Q80K mutation is associated with significantly reduced SVR12 rates. Given the range of other DAAs currently available, most experts would recommend against treating with simeprevir in the presence of this mutation, which is much more common in HCV genotype 1a than 1b (23). More recently, the NS5A inhibitor daclatasvir in combination with other drugs was approved for the treatment of genotypes 1, 2, 3, and 4. In Japan, daclatasvir with the NS3 protease inhibitor asunaprevir was approved for the treatment of genotype 1-infected adults. Notably, although this all-oral combination was the first approved interferon-free regimen and is effective against genotype 1b HCV, baseline resistance-associated variants (RAVs) are very important. In the overall genotype 1b population, SVR rates were 90% with 24 weeks of therapy. However, in those with baseline NS5A RAVs, which limit the activity of daclatasvir, the SVR rate fell to just 40%. With additional research, we may learn that NS5A RAV testing should be a requirement for use of this regimen, particularly given that it is a frequent finding, occurring in 13% to 20% of genotype 1 patients (24).

While resistance to HCV therapies is a concern, unlike those in human immunodeficiency virus (HIV), some baseline HCV drug resistance mutations are often not clearly related to treatment outcome. This is likely due to the higher viral potency of the drugs and their curative nature, which leads to a defined duration of therapy as opposed to lifelong therapy, as is the case with antiretrovirals. HCV treatment failure also differs from HIV treatment outcomes due to the lack of an archived viral reservoir. The majority of patients who fail HCV antiviral therapy with DAAs do so as a consequence of the acquisition of resistance-associated mutations. The importance of the RAVs that emerge likely differs significantly from the importance of those found in HIV infection. With first-generation protease inhibitors boceprevir and telaprevir, long-term follow-up data suggest that resistant variants in most patients were displaced by the wild-type virus as the dominant viral species over time (25, 26).

Although HCV variants (such as S282T, which confers a 9.5-fold reduction in potency) that cause resistance to the NS5B inhibitor sofosbuvir have been identified *in vitro*, very few have been detected in patients relapsing after treatment with sofosbuvir in combination with either ribavirin or a second DAA, likely because of the very poor replicative fitness of this variant. However, failure of treatment of infections caused by variants with resistance to NS5A inhibitors could have long-term clinical implications, as these variants are very fit and appear to persist beyond 2 years of follow-up. Because NS5A inhibitors are important components of almost all approved DAA regimens as well as of those in development, the presence of RAVs with resistance to NS5A inhibitors, which generally leads to cross-resistance across different NS5A inhibitors, may be very important (27). For other NS5A inhibitors in clinical development, certain RAVs have been shown to confer a >5-fold reduction in susceptibility in HCV genotype 1a-infected patients, which dramatically impacts SVR rates (28). In treatment-naive patients, baseline HCV resistance testing should be carefully considered in the context of available therapies if the results would provide physicians with clear therapeutic options. This will not be the case with most treatment-naive patients. In treatment-experienced patients, particularly those with cirrhosis, such testing may be useful. Currently, there is no regulatory-agency-approved assay for the determination of HCV antiviral drug resistance and testing is largely performed in specialized settings with self-validated, laboratory-developed, sequencing-based assays.

## ROLE OF HCV DIAGNOSTIC ASSAYS FOR ON-TREATMENT MONITORING AND POSTTREATMENT AND AS ADHERENCE MEASURES WITH DAA REGIMENS

Apart from some interferon-containing regimens, which rely on HCV load monitoring for assessment of treatment futility rules, decisions with regard to treatment duration with second- and third-generation DAAs are not guided by monitoring for treatment viral kinetics, although diagnostic tests remain critical for the management of patients with HCV. Virologic responses to the newest therapies depend not only on prior treatment experience, including response to previous anti-HCV treatment and other pretreatment clinical indicators, but also on viral genotype and subtype as well as baseline viral load in order to determine the optimal treatment regimen and its duration. The combination of the NS5B inhibitor sofosbuvir plus the NS5A inhibitor ledipasvir has been formulated into a single tablet, which has received approval in Europe and the United States for the treatment of HCV infections. Approval was granted based upon trials that reported SVR rates of >90% (and up to 99%) in HCV genotype 1-infected patients treated with an all-oral, one pill/day regimen. The approved label for this combination indicates that treatment-naive noncirrhotic patients with a baseline viral load of <6 million IU/ml are eligible for as few as 8 weeks of therapy whereas all others should receive a full 12 weeks of therapy. As seen previously with the establishment of RGT rules, no attention was paid during the establishment of this treatment truncation rule to the different performance characteristics of commercially available viral load tests and how they compare to those of the single, manual method used in clinical trials but not widely used in clinical practice. Independent studies designed to evaluate the clinical impact of assay variability and the time of sampling as determinants of treatment truncation for this regimen found that a substantial proportion of patients had fluctuating viral loads above and below 6 million IU/ml at different screening time points that could potentially impact treatment decisions (29). Based on the known bias between quantitative values given by HPS and ART assays and preliminary data from this and other studies, a viral load of 2 million IU/ml with ART could be considered equivalent to 6 million IU/ml with HPS.

The combination ombitasvir/paritaprevir/ritonavir and dasabuvir received approval in Europe and in the United States, with trials describing SVR rates of >95% in the majority of patient populations treated with this regimen for 12 weeks. However, while this regimen is not impacted by baseline viral load, treatment duration and the potential need to utilize ribavirin depend on the HCV genotype 1 subtype and the degree of cirrhosis. Noncirrhotic patients infected with HCV genotype 1b are eligible for 12 weeks of therapy without ribavirin, while HCV genotype 1a infection requires the use of ribavirin and up to 24 weeks of therapy for cirrhotic patients.

On the basis of the approved label for these new combination therapies, HCV geno(sub)type and baseline viral load assessments will continue to be important pretreatment markers that will guide treatment selection and duration. In addition, on-treatment changes in HCV RNA levels continue to be used as a marker of treatment adherence/response and are the measure of treatment success or failure. Recent data illustrated that very early viral kinetics, described as undetectable viral load at early on-treatment time points, was a positive predictor of treatment success and that the more sensitive the test used to measure viral load, the higher the positive predictive value of an undetectable viral load result (30). In contrast, the detection of residual viremia with a very sensitive method even as late as end of treatment (EOT) was not always associated with treatment failure. This would imply that measurement of very early viral kinetics with a very sensitive method may be useful to identify those patients who might benefit from very short treatment durations with highly potent antiviral therapies. In this situation, low levels of residual viremia should not be considered an indication of failure or an indication for treatment extension (21).

Another role for HCV diagnostic testing in the era of DAA-based therapy is as a measure of medication adherence. Patients who are adherent to new, highly potent DAA regimens have been shown to have undetectable HCV RNA very early in therapy. Of note, studies have shown that low levels of quantifiable HCV RNA on therapy and even at the end of treatment should not be considered indicative of nonadherence and do not preclude achieving an SVR. In this context, an accurate but less sensitive method, such as measurement of HCV core antigen levels, may be a valuable alternative to highly sensitive HCV RNA testing as an adherence measure. If a patient receiving a DAA has detectable viremia with a less sensitive method, e.g., a limit of detection (LOD) between 1,000 and 5,000 IU/ml after 4 weeks of therapy, the patient is potentially nonadherent or failing therapy. If confirmed by a more sensitive HCV RNA test, this information could trigger a valuable clinical intervention such as a change in the DAA. Alternatively, analysis of the HCV core antigen levels could be used for this purpose. Undetectable HCV core antigen levels during therapy may be particularly important in patients, such as active substance abusers, in whom adherence may be questioned.

Posttreatment, it is important to continue to monitor for HCV recurrence, particularly among high-risk groups such as active substance abusers and HCV/HIV-infected MSM. Among MSM, the acute HCV reinfection rate ranges from 7.8 to 15.2 per 100 person-years of follow-up (31). Antibodies to HCV persist in the body long after the patient has cleared the infection following either effective therapy or spontaneous resolution. However, achieving an SVR or spontaneous resolution of infection does not prevent subsequent reinfection with the hepatitis C virus. This means that the anti-HCV test routinely used to screen the general population is not useful to identify patients who have been reinfected. For populations who are considered at higher risk for reinfection, such as PWID or MSM, a direct marker for the presence of the virus (RNA or core antigen) should be used to screen these patients for reinfection.

## FUTURE DIRECTIONS AND CONSIDERATIONS OF THE ROLE OF HCV DIAGNOSTIC TESTING

The impact of chronic HCV infection is increasing the burden on global health systems in a variety of ways. Although the HCV epidemic impacts both developed and developing countries, levels of access to diagnostics and treatments are often disparate between geographic areas and even between populations in the same country. Economic considerations will impact the HCV treatment programs adopted by countries as payers and health care systems struggle to deal with competing costs associated with chronic disease and multiple comorbidities in an aging population. Every aspect of the treatment cascade, including diagnostic and on-treatment therapeutic monitoring, must be optimized to maximize positive treatment outcomes. From the efficacy standpoint, future treatment regimens will likely be simpler, without requiring pretreatment assessment of factors that have traditionally influenced response rates such as HCV genotype, IL-28B type, baseline HCV RNA, and the degree of fibrosis. If this simple “one size fits all” treatment paradigm is adopted with new highly potent antiviral regimens, then the diagnostic testing requirements may be limited to the identification of people with active HCV infection, adherence to therapy, and evaluation of treatment success at 12 or 24 weeks posttherapy. If this were to become the case, highly sensitive molecular tests might no longer be necessary, and alternative test methods, which might be less sensitive (potentially qualitative) but which would be less expensive and offer a shorter time to result, might be an option. Two factors, however, weigh heavily on the likelihood of the second scenario becoming reality. First, early real world effectiveness results of treatment with the DAAs appear to be reduced compared with results obtained in clinical trials (32). Second, given the high cost of DAA combination therapy, third-party payers have implemented onerous approval requirements that include determination of HCV genotype and HCV RNA levels and assessment of fibrosis status by biopsy or via noninvasive methods. Additionally, these results may be required to be obtained within a 30-to-90-day period prior to seeking approval, potentially leading to substantial repetition in diagnostic testing, given the multiple layers and extensive documentation requirements of the medication approval process (33).

Future research may find that more-complex strategies are feasible whereby patients are stratified and therapies selected on the basis of the individual patient's likelihood of achieving cure, as measured by pretreatment testing, or potential to undergo progressive fibrosis. As approval of DAA-based therapies is generally limited to cases involving individuals with advanced (stage 3 to 4) fibrosis, accurate identification of those individuals who are likely to undergo fibrosis progression could identify a population subgroup that would be prioritized for antiviral therapy.

Since HCV disease impacts patients in a wide variety of geographic, socioeconomic, and cultural circumstances, diverse approaches to diagnostics and patient care need to be carefully evaluated on the basis of rigorous research of health economics outcomes. One field of HCV diagnostics that has generated considerable interest is point-of-care testing (POCT) for both serology and molecular assays. The ideal POCT is minimally invasive, uses saliva or finger-stick blood as a primary sample, yields results quickly (in minutes) with high accuracy, differentiates individuals who have been exposed to HCV from those actively infected with the virus, is portable and self-contained, providing access to diagnostic testing in areas lacking infrastructure, and is available at a low cost. To date, with the exception of tests using anti-HCV antibodies, no such POCT is currently available.

POCTs are affected by a variety of considerations, including whether testing is done in an urban or rural setting as well as the target population. Population density worldwide is predominantly urban, which would imply that a large proportion of infected patients live in urban areas where access to phlebotomy and sample transport/logistics may not be an issue. Under those circumstances, a rapid, less expensive, centralized test might be a more cost-effective approach. The requirement for testing to be performed in a central laboratory, coupled with the need for phlebotomy followed by refrigeration or freezing of samples until the time of testing, may limit implementation of HCV infection diagnosis and initiation of therapy in rural resource-limited settings and in key affected populations, such as PWID, where venipuncture and cold chain transportation may be difficult.

Additionally, diagnostic testing to determine infection status, particularly in the case of tests that can be performed at the point of care, is particularly important in disenfranchised or marginalized populations such as PWID and MSM. One method that could facilitate HCV infection diagnosis in these population subgroups is the HCV core antigen test, which has a lower limit of detection of approximately 1,000 to 3,000 IU/ml, is usually less expensive than an HCV RNA test, is highly automated, and gives a result in approximately 60 min. Development of the core antigen as a POCT that could be performed on dried blood spots (DBS) would facilitate the diagnosis of HCV infection by permitting testing to be performed in venues where high-risk populations routinely congregate. Such a testing infrastructure could also facilitate communication of test results by potentially eliminating the need for seropositive individuals to return for HCV RNA confirmatory testing.

Platforms such as Cepheid XPERT offer a decentralized solution which moves testing from a centralized reference laboratory to clinics and potentially to the physician's office, providing results in approximately 2 h. This approach is one step removed from the central laboratory but still requires phlebotomized samples and significant infrastructure, features that are missing in many resource-limited settings. Additionally, the platform is not low cost, which might invalidate the claim that this platform can be considered a POCT. The Xpert MTB/RIF test was widely adopted by the South African Ministry of Health in 2011 as the first-line test for detection of Mycobacterium tuberculosis. In the XTEND study, 4,412 results of tests comparing Xpert to acid-fast bacillus (AFB) smear analysis were evaluated. The authors found that there was no difference between the study arms in terms of mortality rates or the numbers of persons starting M. tuberculosis infection therapy. Although Xpert results can be generated in as little as 2 h, results from both the AFB smear and Xpert tests were available to the treating physician in an average of 2 days (34). Decentralized technologies have been associated with high direct and indirect costs and have required significant donor subsidies to facilitate implementation in resource-limited settings, Because of associated costs and issues involving access to established centralized testing, Xpert and other point-of-care solutions have not been widely adopted in developed countries for treatment of infections by blood-borne pathogens.

The use of specimen types other than frozen blood products for HCV diagnostic assays may eliminate this logistical limitation and improve clinical management of patients. In resource-limited settings, the collection of DBS represents an alternative that is minimally invasive, does not require a trained phlebotomist, and has proven effective in the diagnosis and monitoring of patients with HIV-1. HCV proteins and nucleic acids from DBS have been successfully quantified using immunoassays and PCR-based assays (35). The implementation of true point-of-care tests (as defined previously) for HCV infection to screen for new infections as well as to access marginalized patients or patients in remote/rural locations is an attractive prospect.

It is clear that there is a significant lack of the funding to treat everyone infected with viral hepatitis in the world. Under these circumstances, there is an obligation to justify each expenditure by ensuring that it is done in the most cost-effective way to impact the end goals of identifying, treating, and curing as many people as possible, thus avoiding potential disease sequelae and associated health care costs. There are some key questions that should be addressed as health care systems grapple with how best to implement new HCV programs worldwide:

- Using various testing approaches, how many HCV-seropositive patients may be lost to follow-up and what are their demographics? This information may enable such individuals to be targeted more effectively by other testing approaches.
- What are the clinical consequences of an HCV-seropositive patient who is lost to follow-up, given the slow progression of this disease and the low percentage of HCV-positive patients who progress to cirrhosis or HCC before the next testing opportunity?
- What is the likelihood of an HCV-seropositive patient who is lost to follow-up transmitting the virus before the next testing opportunity? How would this vary with population and risk behavior (i.e., PWID versus baby boomer)?

As DAA therapies continue to increase in effectiveness with minimal side effects, will physicians prescribe and third-party payers approve treatment of HCV-infected patients solely on the basis of the diagnostic test result for active HCV viremia or will they require other tests (which may not be available as POCT) such as for HCV genotype, alanine aminotransferase levels, platelet count, or degree of fibrosis (Fibroscan/Fibrotest)?

Diagnostics have played an important role in numerous aspects of HCV, from its discovery to prevention of its transmission, treatment management, and, hopefully, eventual global eradication. The impact of chronic HCV infections is increasing the financial burden on global health care systems. Although the HCV epidemic impacts both developed and developing countries, levels of access to diagnostics and treatments are often disparate between geographic areas and even between populations in the same country. Within developed countries, the changing demographics of the HCV epidemic, which are occurring largely as a consequence of ongoing prescription opiate abuse and transmission through the HIV-infected MSM community, will have an impact on the need for diagnostic testing, since individuals in these subgroups account for the majority of incident HCV infections. These facts, combined with the recognition that up to 75% of HCV-infected individuals in the United States remain unaware of their infection status, indicate that alternative diagnostic approaches, including those that might be performed as POCT or even “self-testing,” could have tremendous utility in maximizing the efficiency of diagnosis. As things stand today, many of the commercially available HCV tests are performed using the same instruments as those used to test for HIV-1. Thus, careful consideration should be given to approaches that would maximize the return on global investments to create a testing infrastructure and algorithm to tackle the challenges presented by this often-sympatric HCV pandemic. Thoughtful, pragmatic, evidence-based approaches to identify the best way to roll out HCV therapies, including the necessary associated diagnostics, are needed. Every dollar spent unnecessarily reduces the amount of resources available to pay for medications required to treat the infection and for the physicians who administer them.
